# Genetic basis of hindlimb loss in a naturally occurring vertebrate model

**DOI:** 10.1242/bio.016295

**Published:** 2016-02-18

**Authors:** Emily K. Don, Tanya A. de Jong-Curtain, Karen Doggett, Thomas E. Hall, Benjamin Heng, Andrew P. Badrock, Claire Winnick, Garth A. Nicholson, Gilles J. Guillemin, Peter D. Currie, Daniel Hesselson, Joan K. Heath, Nicholas J. Cole

**Affiliations:** 1 Department of Biomedical Sciences, Faculty of Medicine and Health Sciences, Macquarie University, Sydney, New South Wales 2109, Australia; 2 Department of Anatomy & Histology, School of Medical Sciences and Bosch Institute, University of Sydney, Sydney, New South Wales 2006, Australia; 3 Walter and Eliza Hall Institute of Medical Research, Parkville, Victoria 3052, Australia; 4 Institute for Molecular Bioscience, University of Queensland, St Lucia, Brisbane, Queensland 4072, Australia; 5 Australian Regenerative Medicine Institute, Monash University, Clayton, Victoria 3800, Australia; 6 Garvan Institute of Medical Research, Diabetes and Metabolism Division, Sydney, New South Wales 2010, Australia; 7 St. Vincent's Clinical School, UNSW Australia, Sydney, New South Wales 2052,Australia

**Keywords:** Pelvic fin, Development, TALENs, Hindlimb, Tbx4

## Abstract

Here we genetically characterise *pelvic finless,* a naturally occurring model of hindlimb loss in zebrafish that lacks pelvic fin structures, which are homologous to tetrapod hindlimbs, but displays no other abnormalities. Using a hybrid positional cloning and next generation sequencing approach, we identified mutations in the nuclear localisation signal (NLS) of T-box transcription factor 4 (Tbx4) that impair nuclear localisation of the protein, resulting in altered gene expression patterns during pelvic fin development and the failure of pelvic fin development. Using a TALEN-induced *tbx4* knockout allele we confirm that mutations within the Tbx4 NLS (A78V; G79A) are sufficient to disrupt pelvic fin development. By combining histological, genetic, and cellular approaches we show that the hindlimb initiation gene *tbx4* has an evolutionarily conserved, essential role in pelvic fin development. In addition, our novel viable model of hindlimb deficiency is likely to facilitate the elucidation of the detailed molecular mechanisms through which Tbx4 functions during pelvic fin and hindlimb development.

## Abstract (precis)

**Summary:** Here we genetically characterise mutations in *tbx4* which underlie *pelvic finless*, a naturally occurring model of hindlimb loss in zebrafish that lacks pelvic fin structures.

## INTRODUCTION

The study of limb development has relied heavily on mouse and chick embryos as models to understand the genetic mechanisms of limb induction, identity and outgrowth. We now describe a unique and viable *pelvic finless* zebrafish model of pelvic fin development and loss in a high-throughput, genetically tractable, model organism. The paired fins of modern fish species and tetrapod limbs share similar gene and protein expression patterns during limb and fin development as the forelimbs and hindlimbs of tetrapods are evolutionarily derived from the paired fins of ancestral fish (Carroll, 1988; Coates, 1994; Don et al., 2013; Mercader, 2007). Due to this conservation, the paired pectoral fins of zebrafish have emerged as an excellent model for dissecting the genetic mechanisms of vertebrate forelimb initiation and early outgrowth (reviewed in Mercader, 2007). Similarly, as the hindlimbs of vertebrates are evolutionarily derived from fish pelvic fins, the pelvic fins of zebrafish provide a relevant and novel model in which to understand early hindlimb development (Don et al., 2013).

In vertebrate limb development, the paralogous T-box transcription family member genes *TBX4* and *TBX5* are highly conserved regulators of limb development. Throughout the development of many organisms, members of the T-box family are expressed dynamically and are vital for many developmental processes. Mutations in the T-box genes cause developmental defects in a range of organisms ranging from *C. elegans* to humans (Papaioannou, 2001). For example, mutations in *TBX5* cause Holt-Oram syndrome which is characterised by skeletal abnormalities in the upper limb and heart defects and mutations in *TBX4* are linked to developmental disorders of the lower limb, such as small patella syndrome (Alvarado et al., 2010; Ballif et al., 2010; Bongers et al., 2001, 2002, 2004; Lu et al., 2012; Mangino et al., 1999; Wang et al., 2010). Whilst there is conflicting evidence as to the ability of *Tbx4* and *Tbx5* to confer limb-type identity (Rodriguez-Esteban et al., 1999; Takeuchi et al., 2003; Minguillon et al., 2005, 2009; Ouimette et al., 2010), *Tbx5* and *Tbx4* have been shown to be crucial for forelimb and hindlimb development, respectively (Ahn et al., 2002; Garrity et al., 2002; Ng et al., 2002; Rallis et al., 2003; Naiche and Papaioannou, 2003; Ruvinsky et al., 2000; Tamura et al., 1999). While there is strong evidence for a crucial role for *Tbx4* in hindlimb development, there is little known about how this transcription factor functions during their development.

Tbx4 and Tbx5 proteins both contain a conserved DNA binding motif known as the T-box domain, and within this domain lies an evolutionarily conserved nuclear localisation sequence (NLS) (Papaioannou, 2001; Collavoli et al., 2003). Interestingly, whilst both proteins contain a conserved NLS, the forelimb paralogue of Tbx4, Tbx5, exhibits varied cellular localisation during organogenesis (Collavoli et al., 2003; Camarata et al., 2006). In the later stages of forelimb development Tbx5 shows dynamic localisation, being localised to both the cytoplasm and the nucleus (Camarata et al., 2006). Despite the evolutionarily conserved role of Tbx4 in hindlimb/pelvic fin development, the mechanism through which Tbx4 functions during limb and fin development remains unknown. Here we show using a unique *pelvic finless* zebrafish model that not only is Tbx4 required for pelvic fin development, but also that the NLS of Tbx4 must be intact for Tbx4 to play its essential role in the induction of the apical ectodermal ridge and the outgrowth of the pelvic fin.

## RESULTS AND DISCUSSION

*Pelvic finless* is a naturally occurring zebrafish strain in which the development of the pelvic fins (the teleost equivalent of hindlimbs) fails (Don et al., 2011). These zebrafish are unique in that only the development of pelvic fins is altered and *pelvic finless* zebrafish are viable as adults as no other structures or developmental processes are affected. *Pelvic finless* zebrafish initiate pelvic fin development, evident by the 3-4 cell thick mesenchymal bulges that form in the pelvic regions around 3-4 weeks of development; however, these bulges do not form an apical ectodermal ridge and a subsequent loss of pelvic fin development is observed (Don et al., 2011).

**Fig. 1.**
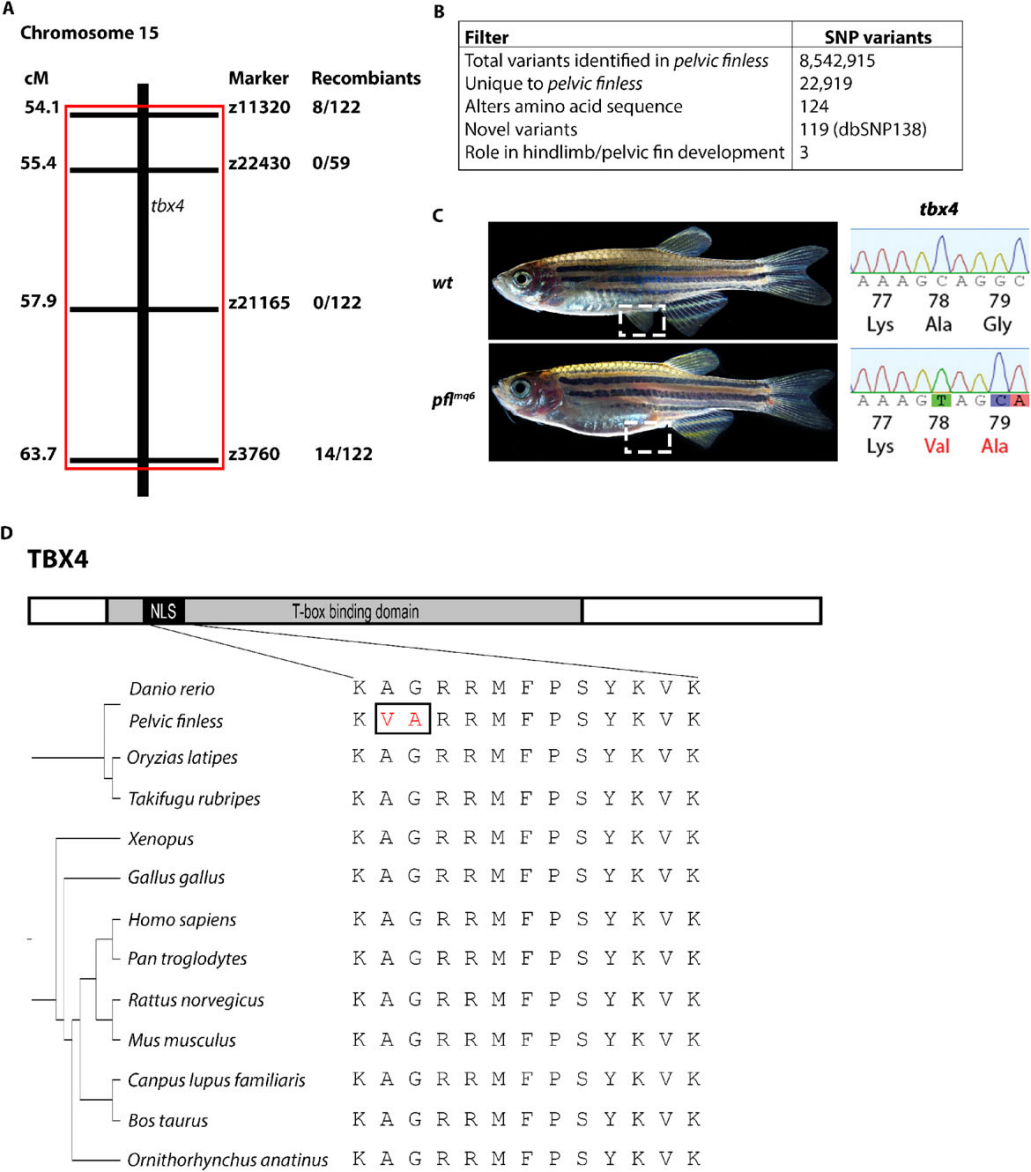
**The *pelvic finless* critical region maps to a region on chromosome 15 containing three SNPs in *tbx4*.** (A) Low and intermediate resolution mapping reveals that the *pelvic finless* critical region lies between 54.1 and 63.7 cM on chromosome 15. (B) Region-specific, targeted-enrichment next generation sequencing filtering process revealed three SNPs in *tbx4*. (C) The three SNPs in exon 3 of *tbx4* of *pelvic finless* zebrafish are predicted to cause two amino acid substitutions (A78V and G79A). (D) Schematic diagram of the conservation of vertebrate TBX4 nuclear localization signal (NLS). The NLS is located within the highly conserved T-box domain of the protein. The sequence of the TBX4 NLS is perfectly conserved amongst vertebrates with hindlimbs or pelvic fins, *pelvic finless* zebrafish exhibit variations in this motif.

We have now determined that polymorphisms in *tbx4* are responsible for the specific and complete absence of pelvic fins in *pelvic finless* zebrafish (Fig. 1A-D). Using genetic mapping, we narrowed the genetic interval containing the *pelvic finless* locus to a 10 cM region of chromosome 15. Region-specific, targeted-enrichment next generation sequencing identified three nucleotide variations in codons 78 and 79 that encode two nonsynonymous amino acid mutations (A78V; G79A) in the NLS of Tbx4 that segregate invariably with the *pelvic finless* phenotype (*n*=122) (Fig. 1A-D).

Alignment of the Tbx4 NLS across all vertebrates for which an entire NLS sequence is available, shows that the motif is perfectly conserved in all species with hindlimbs or pelvic fins (Fig. 1D). Therefore, we hypothesised that the A78V; G79A mutation in the Tbx4 NLS underlies the loss of the pelvic fin development in zebrafish. To confirm whether mutations in the Tbx4 NLS are responsible for the developmental defects in *pelvic finless* zebrafish, we used genetic complementation, protein localization and *in-situ* hybridisation studies to explore the functional consequences of the naturally occurring A78V; G79A mutations.

**Fig. 2.**
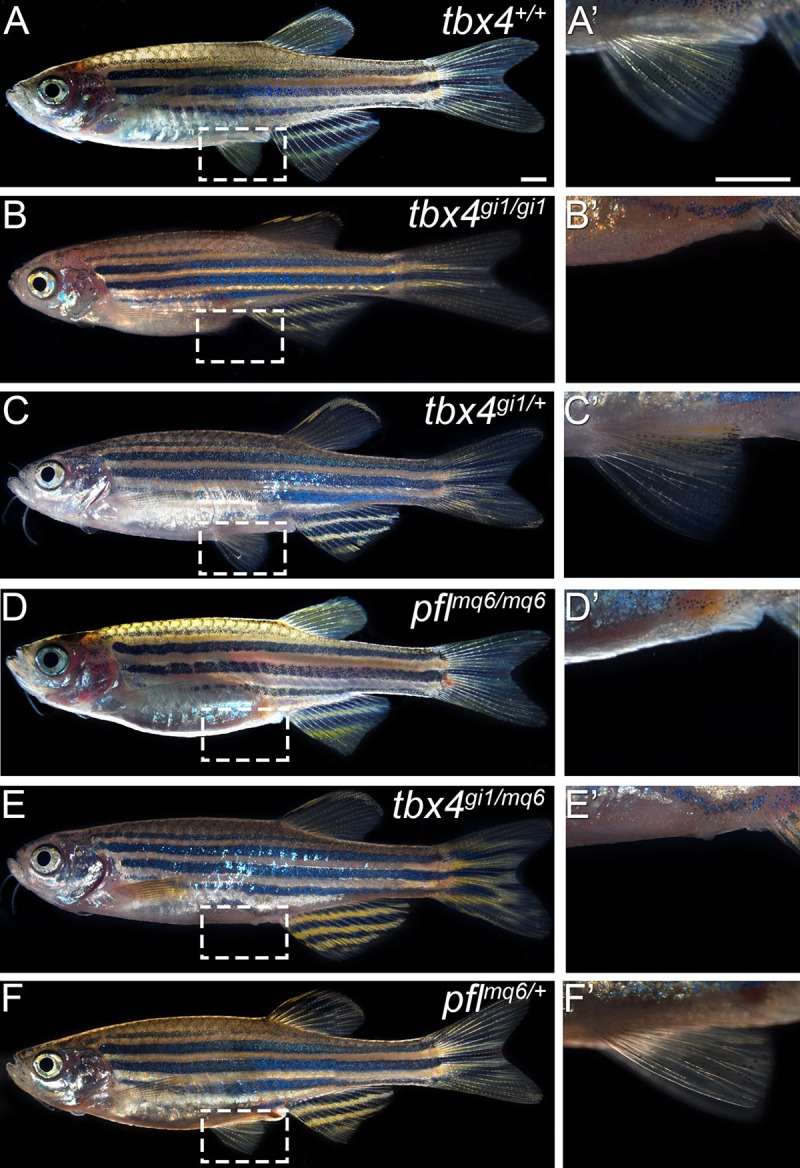
**Mutations in the Tbx4 NLS sequence cause the loss of pelvic fins in *pelvic finless* zebrafish.** (A) Wild-type zebrafish (*tbx4^+/+^*) pelvic fins are located on the posterior ventral flank of the fish, either side of the cloaca. (B) TALENs-induced mutated *tbx4* causes the loss of pelvic fins in homozygous *tbx4* mutation zebrafish (*tbx4^gi1/gi1^*). (C) Heterozygous mutant *tbx4* zebrafish (*tbx4^gi1/+^*) display normal pelvic fin development. (D) *Pelvic finless* zebrafish (*pfl^mq6/mq6^*) do not develop pelvic fins due to missense mutations in the Tbx4 NLS. (E) *Pelvic finless; mutant tbx4* compound heterozygotes (*tbx4^gi1/mq6^*) also do not develop pelvic fins. (F) Heterozygous wild-type; *pelvic finless* zebrafish (*pfl^mq6/+^*) develop pelvic fins, confirming that the *pelvic finless* mutation is allelic to *tbx4* and that the mutations in the NLS of Tbx4 (A78V; G79A) result in a loss of function of the *tbx4* gene. White rectangle in A-F indicates zoomed in region shown in A′-F′. Scale bars: 1 mm.

Using Transcription Activator-Like Effector Nucleases (TALENs) directed to zebrafish *tbx4*, we introduced a frameshift mutation in exon 5 of *tbx4* in wild-type zebrafish. Zebrafish *tbx4* mutants (*tbx4^gi1/gi1^*) are viable and exhibit the identical pelvic fin loss seen in *pelvic finless* zebrafish (*pfl^mq6/mq6^*) (Fig. 2B,D), while heterozygous animals (*tbx4^gi1/+^*) display normal pelvic fin development (Fig. 2C). We next performed a complementation test with the *pelvic finless* and *tbx4* mutants. Compound heterozygotes (*tbx4^gi1/mq6^*) from a cross between homozygous *pfl^mq6/mq6^* and *tbx4^gi1/gi1^* mutants do not develop pelvic fins and display an identical phenotype to *pelvic finless* zebrafish (*pfl^mq6/mq6^*) (Fig. 2E,D), confirming that the *pelvic finless* mutation is allelic to *tbx4* and that the mutations in the NLS of Tbx4 (A78V; G79A) result in *tbx4* loss-of-function. To the best of our knowledge, these findings demonstrate for the first time that Tbx4 is essential for pelvic fin development and that mutations in the Tbx4 NLS are sufficient for pelvic fin loss *in vivo*.

**Fig. 3.**
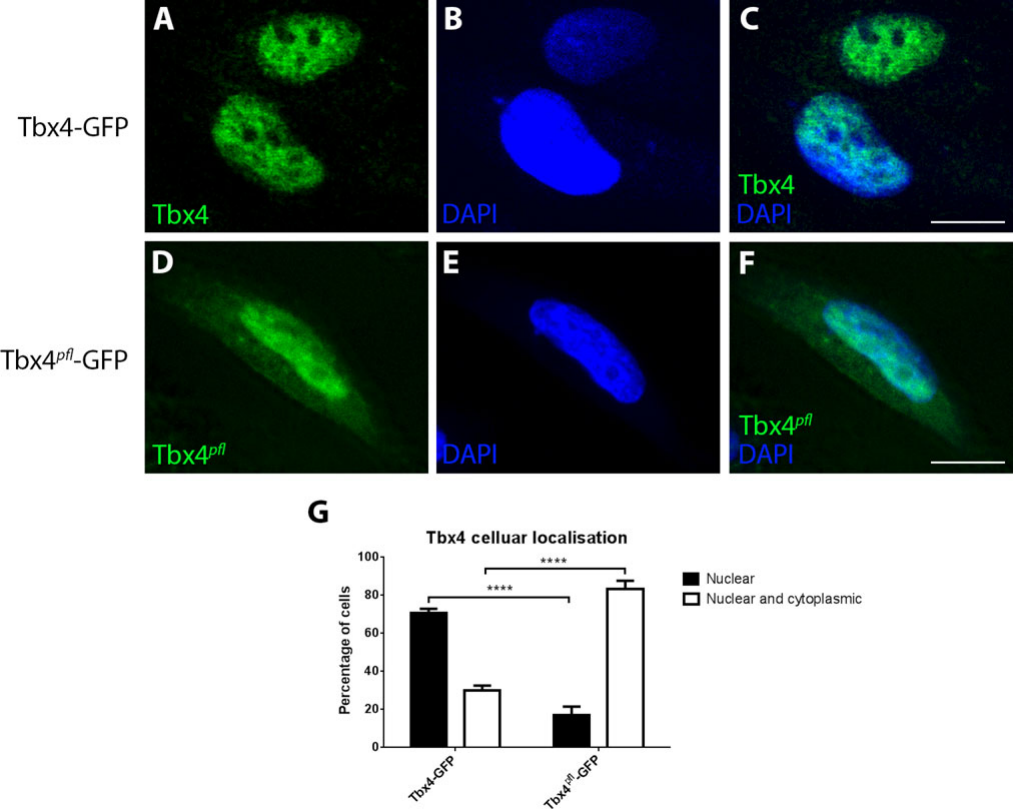
**Mutations in the Tbx4 NLS sequence cause an impairment of nuclear localization of the protein in HeLa cells.** (A-C) Wild-type Tbx4-GFP is located in the nucleus of the majority of cells (70.53±3.9% nuclear only, *n*=110) as shown by co-localisation with the nuclear stain, DAPI. (D-F) Mutations in the NLS of *pelvic finless* zebrafish Tbx4 (Tbx4*^pfl^*-GFP) cause an impairment of nuclear localization of Tbx4 (83.03±7.74% nuclear and cytoplasmic, *n*=83). Scale bars: 10 µm. (G) Graph of the cellular location of zebrafish Tbx4-GFP and Tbx4*^pfl^*-GFP. A two-way ANOVA with Tukey's multiple comparisons test (****P<0.0001). Error bars represent standard deviation from the mean (*n*=3).

We hypothesised that the NLS is essential for Tbx4 function during pelvic fin development as its paralog, Tbx5, requires nuclear localisation to perform its function during limb development (Zaragoza et al., 2004; Fan et al., 2003). To determine whether the A78V; G79A mutation identified in Tbx4 in *pelvic finless* zebrafish compromised nuclear localisation, we transfected C-terminal GFP-tagged Tbx4 constructs into HeLa cells and analysed the sub-cellular localization of the fluorescent proteins by confocal microscopy, due to an absence of specific zebrafish Tbx4 antibodies. We observed wild-type zebrafish Tbx4 (Tbx4-GFP) solely located in the nucleus of the majority of cells (70.53%±3.9% nuclear only, *n*=110) (Fig. 3A-C,G). In contrast, the Tbx4 variant (A78V; G79A) mutated in *pelvic finless* zebrafish (zTbx4*^pfl^*-GFP) shows both nuclear and cytoplasmic localization (83.03±7.74% nuclear and cytoplasmic; *n*=83; Tbx4*^pfl^*-GFP vs Tbx4-GFP, *P*<0.0001) with a proportional reduction in the number of cells which exhibit solely nuclear localisation (Fig. 3D-F,G) suggesting that the conserved Tbx4 NLS sequence facilitates nuclear localisation of the protein. These results suggest that the conserved Tbx4 NLS is required for correct Tbx4 function and that the naturally occurring mutations identified in *pelvic finless* zebrafish impair the function of the NLS, an outcome that is deleterious for Tbx4 function during early pelvic fin development.

**Fig. 4.**
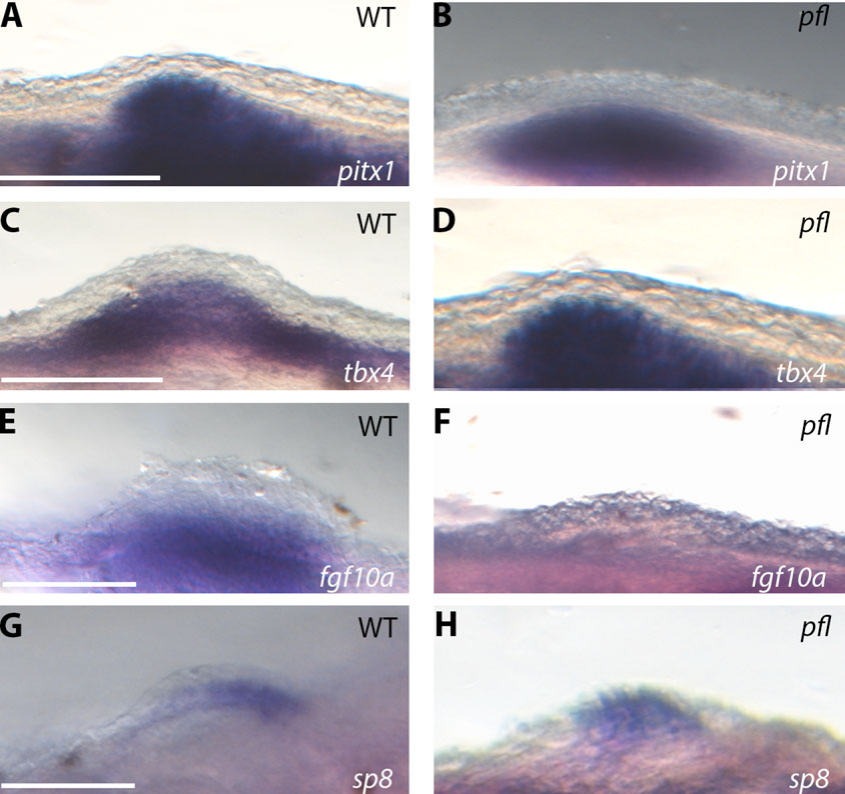
**Mutations in the Tbx4 NLS cause altered expression of pelvic fin outgrowth genes.** (A,B) *pelvic finless* zebrafish express the hindlimb initiation gene *pitx1* in the developing pelvic fin mesenchyme in a similar pattern to wild-type zebrafish. (C,D) *pelvic finless* zebrafish express *tbx4* mRNA in the developing pelvic fin mesenchyme in a similar pattern to wild-type zebrafish. (E,F) There is no detected expression of *fgf10a* mRNA in the developing pelvic fin regions of *pelvic finless* zebrafish, whilst *fgf10a* expression is observed in the developing pelvic fin mesenchyme of wild-type zebrafish. (G,H) Altered expression of the apical ectodermal ridge marker, *sp8*, is observed in *pelvic finless* zebrafish. In *pelvic finless* zebrafish the expression of *sp8* mRNA is not restricted to the apical ectodermal thickening, as seen in wild-types, but is diffuse in the apical ectodermal thickening precursor cells which have failed to accumulate in the dorsoventral boundary in *pelvic finless* zebrafish (*n*=24). Scale bars: 50 µm.

To investigate the downstream consequences of the mutated Tbx4 NLS, we examined gene expression during pelvic fin development in wild-type and *pelvic finless* zebrafish by *in-situ* hybridisation. Expression of early pelvic fin development genes, *pitx1* and *tbx4*, was observed in the mesenchyme of the developing pelvic fin buds of *pelvic finless* zebrafish in a similar pattern to wild-type zebrafish (Fig. 4A-D). We next examined the expression of *fgf10a*, a well characterised direct transcriptional target of Tbx4, which is required to induce and maintain the apical ectodermal ridge during embryonic limb development (Min et al., 1998; Naiche and Papaioannou, 2003). Strikingly, we observed robust expression of *fgf10a* in the pelvic fin region of wild-type zebrafish, but *fgf10a* expression was completely absent from this region in homozygous *pelvic finless* mutants (Fig. 4E,F). In addition, we observed an altered expression of the apical ectodermal ridge marker, *sp8* (Kawakami et al., 2004), in *pelvic finless* fish. Whilst *sp8* expression was observed in the developing pelvic fin apical ectodermal thickening of wild-type zebrafish, *sp8* expression was observed only in the apical ectodermal thickening precursor cells that have failed to accumulate in the dorsoventral boundary of the pelvic fin buds of pelvic finless fish (Fig. 4G,H). Collectively, these data lead us to conclude that mutations in the NLS of Tbx4 impair the function of the Tbx4 protein and compromise its ability to act as a transcriptional activator during early pelvic fin development.

Using a unique *pelvic finless* zebrafish model of hindlimb loss, we demonstrate that Tbx4 has an evolutionarily conserved, essential role in pelvic fin development. *Pelvic finless* zebrafish carrying a naturally occurring mutant version of Tbx4 (A78V; G79A) demonstrate complete pelvic fin loss. Zebrafish harbouring a TALEN-induced mutation in the *tbx4* coding sequence confirm its crucial role in pelvic fin development as these fish also lack pelvic fins. Complementation crosses between these two fish lines demonstrate a striking specificity of pelvic fin loss since we do not observe defects in other structures or organ systems in fish with pelvic fin loss. The essential role for Tbx4 in pelvic fin development has previously been hypothesised, as a result of its crucial role in hindlimb development in mice (Naiche and Papaioannou, 2003) and its pelvic fin expression in zebrafish (Ruvinsky et al., 2000; Tamura et al., 1999). The limb-specific phenotype of our *pelvic finless* zebrafish model will allow for the investigation of the function of Tbx4 and downstream pathways during hindlimb development.

*Pelvic finless* and the TALEN-induced mutated *tbx4* zebrafish described here are novel developmental models in which to examine the cellular function of Tbx4 in the hindlimb/pelvic fin developmental cascade. They will be useful for the functional characterisation of Tbx4 localisation and behaviour during hindlimb development. Because *pelvic finless* zebrafish exhibit specific loss of pelvic fins, with no other defects, *pelvic finless* zebrafish could represent a platform from which to investigate the genetic architecture of hindlimbs or pelvic fin loss in other species. This strategy has been successfully used to investigate the role of *pitx1* (three spine stickleback fish, Cole et al., 2003; Shapiro et al., 2004; Chan et al., 2010) and *hoxd9a* (Fugu, Tanaka et al., 2005) in teleost pelvic fin development.

Our preliminary findings using these novel models show that the evolutionarily conserved Tbx4 NLS is necessary for pelvic fin development as the NLS mutations described in *pelvic finless* zebrafish impede the ability of the protein to function as a transcriptional activator. Our results suggest that the NLS mutations compromise the nuclear localisation of the Tbx4 protein. However, it is also possible that an intact NLS contributes to the DNA binding capacity of the protein. We propose that in the context of early pelvic fin development, the NLS of Tbx4 is necessary for the direct or indirect activation of *fgf10a* to complete pelvic fin bud induction and thus the impairment of Tbx4 NLS leads to pelvic fin loss. Our results suggest that the impairment of the NLS of Tbx4 results in a failure of pelvic fin development due to an inability to establish an apical ectodermal thickening.

Several lines of evidence indicate that the disruption of the apical ectodermal thickening (in fish) or the apical ectodermal ridge (in tetrapods) causes a failure of limb/fin development (Boulet et al., 2004; Crossley et al., 1996; Fischer et al., 2003; Lewandoski et al., 2000; Moon and Capecchi, 2000; Barrow et al., 2003; Narita et al., 2005; Naiche and Papaioannou, 2003; Norton et al., 2005; Min et al., 1998; Sekine et al., 1999). Our findings suggest that during pelvic fin development, the impairment of the Tbx4 NLS results in a loss of *fgf10a* expression, which has been previously shown to result in the failure of the apical ectodermal thickening and the subsequent loss of limb/fin development in multiple animal models (Norton et al., 2005; Min et al., 1998; Sekine et al., 1999). Therefore, in *pelvic finless* zebrafish, impairment of the Tbx4 NLS impedes the ability of the protein to act as a transcription factor, resulting in the loss of the apical ectodermal thickening, and thwarting the development of the pelvic fins.

Our findings are consistent with previous studies of Tbx4 in other systems. Similar to *pelvic finless* or mutated *tbx4* zebrafish, conditional knockout of *Tbx4* in mouse models results in the loss of hindlimbs (Naiche and Papaioannou, 2003, 2007); however early knockout of *Tbx4* results in embryonic lethality in these models. Results obtained from knockout studies of *Tbx5*, the forelimb paralog of *Tbx4*, have also produced similar results. Knockout of *Tbx5* in mice results in the loss of forelimbs (Rallis et al., 2003) and in zebrafish the transient knockdown or knockout of *tbx5* leads to the loss of pectoral fins (Ahn et al., 2002; Garrity et al., 2002; Ng et al., 2002). In humans, mutations in *TBX4* result in the lower limb development defects observed in small patella syndrome, however the mechanism by which the identified mutation cause the lower limb abnormalities remains unknown (Bongers et al., 2004). Mutations identified in *Tbx5* have been shown to cause impaired nuclear localisation of the protein and have been identified as a molecular mechanism responsible for the upper limb and heart developmental defects of Holt–Oran syndrome (Basson et al., 1999; Fan et al., 2003; Li et al., 1997). Seven missense mutations linked to Holt–Oran syndrome (Q49K, I54T, G80R, G169R, R237Q, R237W and S252I) all showed a mislocalisation to the cytoplasm, caused by a nuclear trafficking defect, when transfected into HeLa cells (Fan et al., 2003). Of particular interest, the TBX5G80R mutation, which causes impaired nuclear localisation in Holt–Oram syndrome, corresponds to the Tbx4G79A residue which is mutated in *pelvic finless* zebrafish, suggesting a conserved role for this residue in limb development.

Utilising this naturally occurring vertebrate model of hindlimb loss we have demonstrated specific pelvic fin loss attributed to three single nucleotide variations in *tbx4* cause impairment of the function of the NLS motif in the Tbx4 protein and we have confirmed the sufficiency of these mutations using genetic complementation with a TALEN-induced null allele. Unlike the embryonic lethality of *Tbx4* null mice, the limb specificity of *tbx4* mutations in zebrafish sets the stage of epistatic analysis of the genetic program underlying hindlimb development and is likely to facilitate the elucidation of the detailed molecular mechanisms through which Tbx4 functions in both the cytoplasm and nucleus during pelvic fin and hindlimb development.

## MATERIALS AND METHODS

### Zebrafish

The use and treatment of animals in this project were in accordance with and approved by the Animal Ethics Review Committee, University of Sydney, N.S.W., Australia (ARA: K031-201235665) and the Animal Ethics Committee, Macquarie University, N.S.W., Australia (ARA: 2013-006). Zebrafish (*Danio rerio*) were housed at 28°C, in a 13 h light and 11 h dark cycle. Embryos were collected by natural spawning and raised at 28°C in E3 solution according to standard protocols (Westerfield, 2000).

### Positional cloning

Homozygous *pelvic finless* zebrafish (*pfl^mq6^*) were out-crossed to wild-type WIK zebrafish to generate polymorphic mapping strains. Bulk segregant analysis and rough mapping was performed using the MGH SSLP panel as described (Zhou and Zon, 2011) on 122 *pelvic finless* zebrafish and 40 wild-type siblings.

### Region-specific, targeted-enrichment next generation sequencing and mutation analysis

Genomic DNA was extracted from individual *pelvic finless* and homozygous wild-type zebrafish as described (Gupta et al., 2010). Region-specific, targeted-enrichment was performed by the Beijing Genomics Institute (BGI) on the chromosomal region Ch15:24168360 - 36060966 (Ensembl Zv9), which contained the locus of the gene mutated in *pelvic finless* zebrafish as suggested by the mapping experiments, using hybrid array capture with Roche NimbleGen HD2 11.8 Mb sequence capture array (Roche NimbleGen). Paired end sequencing was performed on a Hiseq2000 platform (Illumina). Raw image files were processed with Illumina basecalling Software 1.7 with default parameters and the sequences of each individual were generated as 90 bp pair-end reads. In the target region, 11,075,935 bp were sequenced to an average depth of 67× with a target region coverage of 95.04% in the *pelvic finless* sample and 93.83% in the wild-type sample. The fraction of unique mapped bases on, or near target was 88.70% for the *pelvic finless* sample and 89.63% for the wild-type sample. The captured region followed a Poisson distribution which revealed that the captured region was evenly sampled. Only mapped reads were used for subsequent analysis.

Sequence reads were generated by the Illumina HiSeq2000 platform and aligned to Zv9 zebrafish genome assembly using SOAPaligner (soap2.21) (Li et al., 2009b) (for subsequent SNP identification) and BWA v0.6.1 (Li and Durbin, 2009) (for insertion and deletion identification). SNP variants were called using SOAPsnp (Li et al., 2009a) and insertions and deletions were identified using GATK (McKenna et al., 2010). All variants were annotated by BGI. Filtering of coding variants was performed using dbSNP (release 138, https://www.ncbi.nlm.nih.gov/SNP/), prioritising by known gene function.

Validation and analysis of the *tbx4* mutations was performed by direct DNA sequencing following PCR amplification of coding exons (ENSDART00000018603). PCR products were Sanger sequenced using Applied Biosystems 3730 and 3730xl capillary sequencers and Big Dye Terminator (BDT) chemistry version 3.1 (Applied Biosystems) under standardised cycling PCR conditions. The raw chromatogram trace files were analysed using Geneious^®^ 6.0.3 software (Biomatters).

### Mutations

The mutations identified in *pelvic finless* zebrafish (*pfl^mq6/mq6^*) were identified as homozygous SNPs in exon 3 of *tbx4* as follows:

mRNA position 233, codon number 78, codon change GCA→GTA, residue change A→V (referred to as A78V in the text),
mRNA position 236 and 237, codon number 79, codon change GGC→GCA, residue change G→A (referred to as G79A in the text).

### TALENs

A pair of TALENs recognising exon 5 (aa121-169) of zebrafish *tbx4* gene was designed using TAL Effector-Nucleotide Targeter and the TAL effector repeats were constructed by the ‘golden gate’ method as described previously (Cermak et al., 2011). TALEN mRNA was synthesised by *in vitro* transcription using the SP6 mMESSAGE mMACHINE Kit (Ambion). 100 pg of mRNA encoding each TALEN heterodimer was injected into the cytoplasm of the cell of one cell-stage wild-type zebrafish embryos. One F1 line (*tbx4^gi1/gi1^*) derived from TALEN injected fish harbours a 7 bp deletion after mRNA position 492 resulting in a frameshift and a premature stop codon at codon position 164. In the text, individuals heterozygous for this mutation are referred to as *tbx4^gi1/+^* and homozygous individuals are referred to as *tbx4^gi1/gi1^* or TALEN-induced mutated *tbx4* zebrafish.

Complementation crosses were performed by crossing a F_0_ *tbx4^gi1/+^* founder harbouring the 7 bp deletion to homozygous *pelvic finless* zebrafish (*pfl^mq6/mq6^*) or homozygous wild-type zebrafish (*tbx4^+/+^*). Homozygous *tbx4^gi1/gi1^* zebrafish were obtained from crosses of a F_0_ *tbx4^gi1/+^* founder to an identified F_1_ *tbx4^gi1/+^* zebrafish. Offspring harbouring the 7 bp deletion were screened at 5 weeks post fertilisation for the presence or absence of pelvic fins. Selected fish were euthanized and imaged in 3% methyl cellulose on a Leica M165FC stereo dissection microscope.

### Cellular localisation

For cellular localisation experiments, cDNAs encoding zebrafish wild-type (Tbx4-GFP) and *pelvic finless* zebrafish (Tbx4*^pfl^*-GFP) C-terminal EGFP tagged sequences were generated by GeneArt (Invitrogen). cDNAs were subcloned into the BamHI and EcoRI sites of pCS2+ (Addgene). All constructs were verified by DNA sequencing.

HeLa cells were cultured in DMEM media (Life Technologies) containing 1% penicillin/streptomycin antibiotics and 10% FBS. Cells were maintained in a humidified 37°C incubator with 5% CO_2_. For transfection, HeLa cells were seeded at a density of 0.3×10^5^ cells/well on poly-L-lysine 35 mm glass bottom culture dishes (MatTek). pCS2+ Tbx4-EGFP plasmids were introduced by transfection into cells using 1 µg of plasmid, 2 µl lipofectamine 2000 (Invitrogen) and 500 µl OPTI-MEM (Invitrogen) according to the manufacturer's protocol. Transfection solution was removed and replaced with complete media with no antibiotics 6 h after transfection. Cells were fixed at 24 h with 4% paraformaldehyde in phosphate buffered saline (PBS) and cover-slipped with Prolong Gold Antifade reagent with DAPI (Invitrogen) to stain nuclei.

Confocal microscopy was performed using a Leica DM6000 upright laser scanning confocal microscope with Leica application suite advanced fluorescence software. Images were acquired with a 40× (1.4 NA) water immersion lens with DAPI and GFP channels using identical settings. Nuclear or cytoplasmic localization data was acquired from five random fields per coverslip. The number of cells with nuclear and/or cytoplasmic localization was counted and presented as a ratio of the total number of transfected cells in a visual field. Data were obtained from three independent experiments in biological triplicates. A two-way ANOVA with Tukey's multiple comparison test was performed to determine significance between samples.

### *In situ* hybridisation

Whole-mount *in-situ* hybridisations were carried out on pelvic regions essentially as described (Westerfield, 2000). Plasmids containing fragments of *fgf10a* and *sp8* (Nagayoshi et al., 2008), *fgf8a* (Komisarczuk et al., 2009), *tbx4* (Tamura et al., 1999) were kindly donated for use in this project. A fragment of *pitx1*was amplified using primers (Forward: 5′GGACTCACTTCACNAGCCAGCAG, Reverse: 5′TAGGCTGGAGTTGCAVGTGTCCCGGTA) and cloned into pCR^®^4-TOPO^®^ vector. Digoxigenin-labelled riboprobes were generated with SP6, T3 or T7 RNA polymerases (Roche) according the manufacturer's instructions. Post staining, pelvic fins were dissected and mounted in 3% methyl cellulose and imaged using a Leica M165FC stereo dissection microscope. All experiments were performed in triplicate on pooled individuals (*n*=24) from multiple spawnings.

Gene sequences were obtained from Ensembl. Database accession numbers are as follows: Human (*Homo sapiens*: ENSG00000240335); Chimpanzee (*Pan troglodytes*: ENSPTRG00000009491); Mouse (*Mus musculus*: ENSMUSG00000000094); Rat (*Rattus norvegicus*: ENSRNOG00000003544); Dog (*Canis lupus familiaris*: ENSCAFG00000017740); Cow (*Bos taurus*: ENSBTAG00000009968); Platypus (*Omithorhynchus anatinus*: ENSOANG00000011525); Chicken (*Gallus gallus*: ENSGALG00000005285); Xenopus (*Xenopus tropicalis*: ENSXETG00000010718); Fugu (*Takifugu rubripes*: ENSTRUG00000008071); Medaka (*Oryzias latipes*: ENSORLG00000014806); Zebrafish (*Danio rerio*: ENSDARG000- 00030058).
